# An update on COVID-19 for the otorhinolaryngologist – a Brazilian Association of Otolaryngology and Cervicofacial Surgery (ABORL-CCF) Position Statement^☆^

**DOI:** 10.1016/j.bjorl.2020.04.002

**Published:** 2020-04-11

**Authors:** Joel Lavinsky, Eduardo Macoto Kosugi, Eduardo Baptistella, Renato Roithmann, Eduardo Dolci, Thais Knoll Ribeiro, Bruno Rossini, Fabrizio Ricci Romano, Rebecca Christina Kathleen Maunsell, Edson Ibrahim Mitre, Rui Imamura, Adriana Hachiya, Carlos Takahiro Chone, Luciana Miwa Nita Watanabe, Marco Aurélio Fornazieri, Marcus Miranda Lessa, Geraldo Druck Sant’Anna

**Affiliations:** Associação Brasileira de Otorrinolaringologia e Cirurgia Cérvico-Facial (ABORL-CCF), São Paulo, SP, Brazil

**Keywords:** Coronaviruses, Otolaryngologist, ENT disease, Coronavírus, Otorrinolaringologista, Doença ORL

## Abstract

**Introduction:**

We are facing a pandemic with a great impact worldwide, as a result of the rapid spread of the novel coronavirus (COVID-19). The medical community is still getting to know behavior of this virus and the consequences from a population point of view. All this knowledge is extremely dynamic, so some behaviors are still not well established. Otorhinolaryngologists have a central role in the management of this situation, in which they must assess the patient, avoid contamination to and by health professionals and other patients. Thus, the recommendations of the Brazilian Association of Otorhinolaryngology and Cervical-Facial Surgery (ABORL-CCF) have the main objective of reducing the spread of the new coronavirus during otorhinolaryngological care and assisting in the management of these patients.

**Methods:**

Review of the main recommendations of national and international scientific societies, decisions by government agencies and class councils. The topics will be related to the general aspects of COVID-19, personal protective equipment, care in patient assistance, endoscopic exam routines and the management of sinonasal, otological and pediatric evaluations related to COVID-19.

**Results:**

The use of personal protective equipment is considered crucial in routine ENT care. We recommend postponing appointments, exams and elective surgeries to reduce the spread of COVID-19. Similarly, we recommend changing routines in several areas of otolaryngology. Additionally, guidance is provided on the use of telemedicine resources during the pandemic period.

**Conclusions:**

We are still at the beginning of the COVID-19 pandemic and scientific evidence is still scarce and incomplete, so these ABORL-CCF recommendations for otorhinolaryngologists may be updated based on new knowledge and the pattern of the new coronavirus spread.

## Resumo

**Introdução:**

Estamos diante de uma pandemia de grande impacto mundial como resultado da rápida propagação do novo coronavírus, COVID-19. A comunidade médica está ainda conhecendo o comportamento desse vírus e as repercussões do ponto de vista populacional. Todo esse conhecimento é extremamente dinâmico, por isso algumas condutas ainda não estão bem estabelecidas. O otorrinolaringologista tem um papel central no manejo dessa situação em que deve avaliar o paciente e evitar a contaminação dos profissionais da saúde e dos demais pacientes. Dessa forma, as recomendações da Associação Brasileira de Otorrinolaringologia e Cirurgia Cérvico-Facial (ABORL-CCF), têm por objetivo principal reduzir a propagação do novo coronavírus durante o atendimento otorrinolaringológico e auxiliar no manejo desses pacientes.

**Método:**

Revisão das principais recomendações das sociedades científicas nacionais, internacionais, decisões de órgãos governamentais e de conselhos de classe. Os tópicos serão relativos aos aspectos gerais do COVID-19, equipamentos de proteção individual, cuidados no atendimento ao paciente, as rotinas dos exames endoscópicos e o manejo de aspectos nasossinusais, otológicos e pediátricos relacionados ao COVID-19.

**Resultados:**

É considerado crucial o uso de equipamento de proteção individual no atendimento otorrinolaringológico de rotina. Recomendamos postergar atendimentos, exames e cirurgias eletivas para diminuir a propagação do COVID-19. Da mesma forma, recomendamos mudança de rotinas em diversas áreas da otorrinolaringologia. Além disso, orientações sobre o uso do recurso da telemedicina durante o período de vigência da pandemia.

**Conclusões:**

Estamos ainda no início da pandemia do COVID-19 e as evidências científicas são ainda escassas, por isso essas recomendações da ABORL-CCF para os otorrinolaringologistas podem sofrer atualizações baseadas nos novos conhecimentos e no padrão de disseminação do novo coronavírus.

## Introduction

Due to the Public Health Emergency of International Importance established on January 30, 2020 by the World Health Organization (WHO)^1^, ^2^ caused by the novel coronavirus and the confirmation of cases of the disease of the new coronavirus in the national territory,^3^ the Brazilian Association of Otorhinolaryngology and Cervico-Facial Surgery (ABORL-CCF, *Associação Brasileira de Otorrinolaringologia e Cirurgia Cérvico-Facial*) has decided to go publish guidelines aiming at updating and guiding otorhinolaryngologists. These recommendations are based on current knowledge: further updates may be required as this pandemic evolves.

## About Coronavirus

Coronaviruses belong to a family of relatively common respiratory viruses, being a frequent cause of the common cold, second only to rhinovirus. In the past few decades, they have been linked to more severe outbreaks, such as the Severe Acute Respiratory Syndrome (SARS) of 2002 and the Middle East Respiratory Syndrome (MERS) of 2012. On December 31, 2019, there was a warning to WHO that several pneumonia cases were occurring in the city of Wuhan (Hubei, China), which were later associated with the new coronavirus strain.

Seven human coronaviruses have been identified: the most common Alpha coronavirus 229E and NL63 and Beta coronavirus OC43 and HKU1; those responsible for the aforementioned SARS-CoV and MERS-CoV outbreaks; and now the new coronavirus, initially named 2019-nCoV, later changed on February 11, 2020 to SARS-CoV-2, as it is genetically related to SARS-CoV. The disease caused by the new coronavirus was named COVID-19.

## About the transmission

The virus transmission occurs from person to person, through respiratory droplets or contact. Anyone who had close contact (approximately 1 m) with someone infected can be exposed to the infection. Given the particularity of the consultations at otorhinolaryngological offices, with the performance of specific physical and endoscopic exams that can generate respiratory droplets, otorhinolaryngologists are at risk for infection.

## About the symptoms

The main symptoms related to COVID-19 are fever, cough, dyspnea and fatigue. It is important to emphasize the possibility that the individual is an asymptomatic carrier. Some regions of the world are more affected than others, further increasing the need for professional care. It is noteworthy to mention other symptoms that may also be present, such as anosmia and taste alterations.

## About personal protective equipment (PPE)

During outpatient visits, we recommend wearing a surgical mask, eye protection, long-sleeve gown and gloves and that these personal protective equipment be used for all consultations.

During endoscopic otorhinolaryngological exams, we recommend using a N95 mask, PFF2 or higher, eye protection, long-sleeved gown and gloves. Likewise, personal protective equipment must be worn in all otorhinolaryngological exams.

## About medical consultations

The Brazilian Medical Association (AMB, *Associação Médica Brasileira*) released a note on March 19, 2020 recommending the suspension of elective outpatient care throughout the country, as well as the postponement of elective surgeries, if possible. On the following day, the Federal Council of Medicine (CFM, *Conselho Federal de Medicina*) reinforced the recommendation of suspending elective medical consultations, but considered that if this is not possible, doctors can carry them out as long as they are in accordance with the determinations of the local authorities and the Service Technical-Director, respecting the recommended rules of hygiene, individual protection and contact restriction.

The otorhinolaryngologist is at the forefront of care for acute respiratory infections and we also understand that our patients will continue to have other diseases with specific demands and treatments that cannot be postponed, such as recent postoperative or oncological diseases. Therefore, the recommendation is to restrict the number of elective face-to-face consultations, maintaining only the treatment of patients with diseases whose treatment cannot be postponed during this crisis period. We advise performing a telephone screening of patients with scheduled consultations or those who request elective appointments. Patients with fever, cough, sudden anosmia and/or flu-like symptoms without dyspnea should be instructed to undergo home isolation for 14 days. Patients with dyspnea or severe symptoms, on the other hand, should be instructed to seek emergency care in referral hospitals. If the doctor and the patient agree, elective consultations during the COVID-19 crisis may be carried out in the ways provided by Telemedicine, according to the new resolution by CFM and the Ministry of Health (described in detail in the section on telemedicine). In case of elective consultations that cannot be postponed, we suggest scheduling appointments at longer intervals between patients, in order to avoid the crowding of people at the reception or waiting room. In the absence of face-to-face care during this period, we suggest that doctors, if possible, offer a communication channel with patients, which allows for adequate recommendations.

## About care at the unit reception

It is important that some guidelines related to patient care be followed, such as asking about the presence of fever, cough, dyspnea and sneezing upon patient arrival. We recommend offering a surgical mask to patients with these symptoms.

Employees at the office reception should also wear surgical masks in this situation and clean their hands thoroughly with soap and by rubbing them with an alcohol-based formulation (gel alcohol or solution) frequently. We advise keeping the reception well ventilated and providing dispensers with alcohol-based formulations (in gel or solution) and tissues in places with easy access for patients and companions.

We recommend providing conditions for simple hand hygiene: sink with liquid soap dispenser, paper towel holder, paper towels, and trash cans with lids that open without manual contact. Clean and disinfect frequently touched objects and surfaces with 70% alcohol, sodium hypochlorite solution or another disinfectant indicated for this purpose. We recommend that pamphlets or posters be made available on the respiratory etiquette: when coughing or sneezing, cover your nose and mouth with your flexed elbow or with a tissue and discard it after use; after coughing or sneezing, wash your hands with soap and water or gel alcohol; avoid touching eyes, nose and mouth without adequate hand hygiene.

## About care during the consultation with an otorhinolaryngologist

It is important to highlight that many infectious patients are asymptomatic or oligosymptomatic and, therefore, the use of personal protective equipment (PPE) is strongly recommended to treat all patients. The use of the above-mentioned PPE should not lead to the neglect of basic respiratory hygiene care, especially hand hygiene. Do not circulate through the office wearing PPE.

We recommend making dispensers with alcohol-based formulations (in gel or solution) available in places that are easily accessible to doctors and patients. Clean and disinfect frequently touched objects and surfaces, with alcohol 70%, sodium hypochlorite solution or other disinfectant agent indicated for this purpose, in addition to performing standard disinfection procedures for examination material.

## About endoscopic otorhinolaryngological examinations

Here are the recommendations related to endoscopic otorhinolaryngological examinations (nasal videoendoscopy, videolaryngoscopy, video-laryngeal stroboscopy, video-nasofibrolaryngoscopy, video endoscopy of deglutition and other functional evaluations).

During this pandemic period, avoid conducting elective tests and make sure that the test is absolutely necessary at the moment and should not be postponed.

Keep the environment ventilated, allowing the dispersion of aerosols to the external environment.^3^ Consider using vasoconstrictors and topical anesthetics to reduce the chance of coughing or sneezing, which can generate aerosols and remain in suspension for a longer period than the droplets.^4^, ^5^ Despite the uncertain epidemiological role, the possibility of SARS-CoV-2 transmission by aerosols has been recently demonstrated.^6^, ^7^, ^8^ Change gloves for each patient and rub alcohol gel on the hands after the procedure. The endoscopy should, if possible, be performed with video documentation to maintain some distance from the patient; it is recommended to avoid direct viewing through the optical system and touching surfaces during the exam. There should be no patient companion in the room, unless it is strictly necessary.

The processing of the materials must follow the ABORL Operation Protocol available at https://www.aborlccf.org.br/imageBank/Manual-POP.pdf, or high-level disinfection with immersion in disinfectant agents according to RDC N. 6 of March 01, 2013. Finally, use 70% alcohol, sodium hypochlorite solution or other disinfectant indicated for this purpose on the entire surface near the patient, in equipment and containers that may possibly be contaminated (e.g., anesthetic or decongestant container).

## About the sinonasal aspects

Viral upper respiratory tract infections (URTIs) are the second leading cause of anosmia, with spontaneous recovery in most cases. A recent study reported only 5.1% anosmia in patients with COVID-19.

However, anecdotal evidence of anosmia in 30% of COVID-19 patients in Daegu, South Korea, and 2/3 of COVID-19 patients in Heinsberg, Germany, alerted doctors to the possibility of anosmia being an alarm symptom for COVID-19. Although there is no robust evidence, we advise that the presence of sudden anosmia (with or without ageusia and without concomitant nasal obstruction) may suggest the occurrence of COVID-19 in this scenario of pandemic and sustained transmission of the SARS-CoV-2 virus, and suggest that patients in these conditions should be instructed to undergo home isolation for 14 days and wait for the resolution of anosmia, which seems to be temporary in most cases.

In line with the current positions of the World Health Organization and the North-American Centers for Disease Control and Prevention, we advise avoiding the use of systemic corticosteroids for the treatment of patients with flu syndrome while the COVID-19 pandemic is in effect. Regarding the use of topical nasal corticosteroids, the current evidence shows no harm and its use can be maintained in patients who have already used this medication chronically under medical advice. However, due to the lack of conclusive studies in relation to COVID-19 and extrapolating from the recommendations of systemic corticosteroids, we advise that chronic topical nasal corticosteroid use be maintained and continue to be indicated, and in the occurrence of fever or other symptoms suggestive of flu syndrome, the doctor may consider temporary interruption. For the use of topical nasal corticosteroids in acute viral infections, there is a conflicting recommendation from the American (2016) and European (2020) Guidelines, so we advise avoiding the use of topical nasal corticosteroids in acute viral conditions in this context of COVID-19.

Regarding nasal irrigation with saline solution (NISS), there is no scientific evidence on the benefits or harms of its use in COVID-19. In patients with COVID-19, as well as other viral URTIs, the use of NISS can be beneficial for symptomatic relief, removal of secretions and prevention of secondary bacterial complications, such as acute rhinosinusitis, being considered an option (and not a recommendation) by the American (2016) and European (2020) Guidelines. However, it was disclosed that the NISS could facilitate the entry of the SARS-CoV-2 virus into the lower airway or that it could spread the virus through the environment, but without scientific evidence to support it. Therefore, we recommend that chronic NISS use be maintained and continue to be indicated. The indication of NISS in acute infectious conditions should be assessed on a case-by-case basis in this context of COVID-19, as it is considered an option by the guidelines. However, we reinforce the need for adequate hygienization of the hands, nasal irrigation instruments and the environment in which the NISS is to be performed. Regarding the sinonasal endoscopic surgeries, especially those using drills or microdebriders, there have been reports of infection of the entire team in the room by a patient with COVID-19 in China, even with the use of adequate protective clothing and N95 masks. Therefore, in accordance with the CFM, we recommend not performing nasal or sinonasal surgeries in the context of the COVID-19 pandemic. In urgent cases or in case of extreme necessity to perform the surgery, we suggest performing the test to identify the new coronavirus (COVID-19) using the new 24-hour test. In positive cases for COVID-19 or if it is impossible to perform the test, one should wear PPE with powered, air-purifying respirators.

## About the otological aspects

As there is an apparent preference of the coronavirus for the upper airway mucosa, which is also present in the middle ear mucosa, there is an increased risk of contamination by the coronavirus in otological surgeries and procedures.^9^, ^10^, ^11^ Although the main route of transmission of the COVID-19 virus is through the respiratory system, there is some evidence of transmission through blood, although this risk is likely to be low. Previous publications have already demonstrated the presence of other types of coronaviruses in the middle ear in cases of acute infection. We do not presently know whether the middle ear mucosa and mastoid cells are affected by COVID-19. Considering the intense involvement of the nose and rhinopharynx, which can potentially lead to middle ear contamination via the auditory tube, in addition to previous evidence of other types of coronaviruses present in the middle ear during upper airway infections, it is plausible to consider the contamination of these structures by COVID-19.^12^

The formation of aerosols due to the use of surgical drills should also be considered and, if the virus is present, it can infect everyone in the operating room, by maintaining a contaminating closed environment for hours.^13^, ^14^ Although the masks prevent the inhalation of particles, standard eye protection may not adequately prevent the surgeon's eye exposure. Thus, otological procedures, including aspiration and mastoidectomy, must be considered as having a high risk of contamination.

The following are considered otological emergencies, requiring immediate surgical procedure: acute complications of diseases of the middle ear with a risk of death (intra-cranial abscesses and otogenic meningitis), and the presence of a foreign body in the ear (batteries, due to the risk of chemical leakage) and malignant temporal bone tumors.

Mastoiditis and complications of middle ear diseases without improvement after clinical treatments, traumatic peripheral facial paralysis or secondary to middle ear disease (acute otitis media and cholesteatoma) without improvement with clinical treatments and ear trauma are considered urgencies and may require surgical programming within 72 h.

Otogenic extracranial abscesses (subperiosteal abscess) should be treated clinically and preferably punctured, avoiding major surgical procedures, except if there is evidence of greater risk of complications. For acute mastoiditis, curettage should be performed whenever possible, instead of using drills. If the use of drills is essential, it is necessary to reduce the rotation to the minimum possible and use powerful and adequate suction to reduce aerosolization.

Vestibular schwannoma surgery should not be considered urgent, unless there is potentially fatal brainstem compression. A retrosigmoid approach, and not a translabyrinthine one, should be used to minimize drainage time and exposure to the middle ear mucosa.

For cases of benign otological neoplasia, perilymphatic fistula due to barotrauma and post-meningitis cochlear implant indication, there is the possibility of surgical postponement for up to 30 days without major damage, always with specialized medical monitoring. Some otological neoplasms can wait up to 3 months without worsening of the prognosis.

The other surgical procedures that do not show a worsening of the prognosis due to postponement, such as treatment of uncomplicated cholesteatoma, tympanoplasty with or without ossicular reconstruction, middle ear implants and bone-anchored prostheses, cochlear implants (except for urgent indications, temporal bone fracture and children with prelingual deafness at risk of worsening the prognosis), vestibular surgeries and ventilation tubes can be postponed for more than 3 months, always with the recommendation of alert monitoring by the otolaryngologist.

The recommendation is that, whenever possible, mastoidectomies should be avoided due to the high risk of aerosol spread and contamination of surgical teams. If the procedure is absolutely necessary, it should be considered as if the patient was positive for COVID-19, due to the impossibility of carrying out tests on all patients and the high possibility of false negatives, and powerful aspirators with a filtering system must be used.

In most otological surgeries (although not desirable) the performance of the operation by the main surgeon only is feasible (foreign body removal, drainage of abscesses/mastoiditis, myringotomy with or without placement of a ventilation tube, myringoplasty, tympanoplasty, and even mastoid antrostomies), minimizing the exposure of other medical colleagues and other health professionals. Of course, it is not the ideal situation in surgical procedures, but the current situation requires the least possible exposure of professionals, on an exceptional basis. When ear surgery is urgent or essential, it should be preferably performed by the most experienced ear surgeon available at the service.

Regarding the use of corticosteroids for the treatment of Sudden Deafness and for Meniere's disease, it is recommended avoiding systemic use, due to the high risk of prognosis worsening in patients infected with COVID-19, even if they are asymptomatic. If necessary, careful use of intra-tympanic corticosteroids should be preferred, as they have much lower systemic absorption, but there are still no studies demonstrating the safety of this application in patients with COVID-19. Thus, it is recommended explaining it clearly to the patient, showing risks and benefits, and requesting the patient's signed consent. Contrary to what is usually indicated regarding the use of intratympanic corticosteroids, the patient must be instructed not to spit saliva to avoid the dispersion of aerosols containing viruses.

For non-traumatic peripheral facial paralysis, especially Bell's, there have been studies that showed an improvement of 85–96% of cases with the use of systemic corticosteroids against a worse prognosis due to non-use.^15^ In these cases, whenever possible, the test for COVID-19 and treatment with corticosteroids should be carried out, if the test is negative. Still, it is recommended explaining it clearly to the patient, showing the risks and benefits, and requesting the patient's signed consent. In cases of necrotizing external otitis, it is believed that COVID-19 infection should not affect the treatment with intravenous antibiotics, but hospital discharge is recommended within the shortest possible period, followed by ambulatory or at-home treatment.

## About the pediatric aspects

Infected children are usually asymptomatic and, when symptoms are present, they have fever, dry cough and fatigue, with few having upper respiratory symptoms, including nasal congestion and rhinorrhea. Some patients have experienced gastrointestinal symptoms, including abdominal discomfort, nausea, vomiting, abdominal pain and diarrhea. Therefore, most infected children have mild clinical manifestations and a good prognosis, thus becoming possible vectors of COVID-19. Consequently, we must consider all children as potential carriers of COVID-19.

For this reason, we recommend that the child's oropharynx be examined only if it is essential for the clinical diagnosis or can lead to a change in the therapeutic approach. It is recommended, at the moment, carrying out as few tests as possible and avoiding repetition, restricting their performance to imminent situations of respiratory failure risk. If a laryngotracheoscopy and/or bronchoscopy is necessary in the operating room due to suspected lesions below the vocal folds, do not perform nasofibrolaryngoscopy at the bedside in wards and intensive care units, where there will be dispersion of aerosols, with a greater number of exposed individuals.^16^

In the case of services with residents or fellows, the cases should be discussed, and the exams anticipated to be carried out according to the norms and with the use of complete PPE. At the time of the examination, it is important that as few people as possible are present in the room (health professionals and the child's family members). When the endoscopic examination is performed in a suspected or confirmed case of COVID-19, the procedure room must undergo terminal cleaning. Hence the importance of testing before the procedure, if possible. Consider the possibility of other complementary exams to elucidate the diagnosis, and exams such as ultrasounds and CT scans should be preferred, particularly for suspected neoplasms and abscesses.

In cases of pharyngotonsillitis, the oroscopy is recommended only if essential for clinical diagnosis. The prescription of antibiotics in children over 3 years of age is recommended if there was a picture of odynophagia and fever in the previous 24 h, in the absence of cold symptoms (cough and runny nose) associated or not with painful adenomegaly.^17^

In subperiosteal abscesses (orbital complications of acute rhinosinusitis), after the implementation of clinical measures, if there is a risk of visual impairment, drainage through external access is recommended whenever possible.

In relation to foreign bodies, especially batteries in any site, removal is indicated. Foreign bodies in the nose, pharynx and airways should be removed as usual, given the possibility of short-term complications, particularly in the case of respiratory obstructions.

In cases of respiratory failure, consider performing an endoscopic examination when it is essential for the diagnosis and it has an impact on patient treatment responsiveness and discharge. The child must have, in addition to stridor, the following signs or symptoms of severity: a fall in oxygen saturation, cyanosis, apnea. Examples: suspected severe laryngomalacia, bilateral vocal fold paralysis, bilateral choanal atresia, laryngeal membrane, neoplastic obstruction, post-intubation obstruction after maximized clinical treatment and 2 extubation failures, emergency difficult intubation. The other situations should be discussed on a case-by-case basis with the emergency medicine colleague. In some situations, the patient may not show signs of severity at the moment, but with an imminent risk that will prevent discharge. Examples: foreign bodies in the airway or patients with previous known diseases, such as laryngeal stricture acquired during endoscopic treatment (dilations) and recurrent laryngeal papillomatosis. In these cases, in hospitalized patients or those with acute symptoms that will be taken to the operating room, we suggest that the COVID-19 testing or the viral profile be done beforehand, whenever possible. In the latter cases, if the child has a tracheostomy, endoscopic examination should be postponed until the end of the pandemic. In patients with post-extubation laryngitis with two extubation failures after clinical treatment, an endoscopic evaluation should be performed in the operating room (diagnostic and therapeutic laryngotracheoscopy). According to the lesion severity and the patient's clinical condition, consider performing a tracheostomy at the same surgical time.

In the management of dysphagia, individualized decision-making is suggested, taking into account whether the symptoms justify the examination to rule out anatomical alterations at the moment and whether the examination at the current moment will change the conduct in the following days or weeks.

## About the performance of tracheostomies

With the progressive increase in the number of COVID-19 cases, it is expected that many patients will require orotracheal intubation and prolonged mechanical ventilation. In this context, the need for a tracheostomy can be considered by the care teams. Its indications, benefits and risks to the patient and the surgical team must be discussed among the involved teams.

In severe cases that require invasive ventilatory support, orotracheal intubation is the initial choice in patients with COVID-19.^18^ In case of emergency surgical access to the airway due to intubation difficulty, a situation that should always be anticipated to allow adequate action in case it is necessary, a cricothyroidotomy is recommended,^19^ surgical or by puncture, followed by a tracheostomy as soon as possible after airway stabilization. In these emergency cases, the same precautions mentioned below must be taken for tracheostomy.

In the pediatric age range, emergency situations with difficult intubation should be anticipated and respiratory failure quickly identified, being the most frequent cause of cardiorespiratory arrest in children. Children expected to require surgical access to the airway should preferably be managed in a surgical environment, with an intravenous access that allows adequate airway management and hyperoxygenation, with positive-pressure ventilation through a face mask, with or without the aid of an oropharyngeal cannula for stabilization. In the case of patients with difficult ventilation and intubation, a laryngeal mask may be used temporarily and, if available, bronchoscopy-guided intubation. In these cases, one should follow the same guidelines for PPE use. Puncture cricothyroidotomy indication in children is extremely rare, which allows oxygenation, but not ventilation. Current APLS (Advanced Pediatric Life Support) guidelines indicate the use of needle cricothyroidotomy in children older than 5 years. In children under 1 year of age, tracheostomy is recommended; and from 1 to 5 years old, cricothyroidotomy or surgical tracheostomy.

The moment of elective tracheostomy indication in patients with prolonged orotracheal intubation is a controversial subject. In these cases, the tracheostomy is considered to prevent laryngotracheal stenosis, to accelerate weaning from mechanical ventilation and to facilitate the cleaning of respiratory secretions. Elective tracheostomy can be indicated from the 4th to the 21st day, most commonly between 10 and 14 days of intubation.

In the pediatric age group, tracheal intubation is better tolerated and the ideal time to indicate a tracheostomy has not been well established, although some authors suggest that, if there is no prospect of weaning from mechanical ventilation after 2 weeks, it should be considered. When maintaining prolonged intubation, care should be taken to use tubes of the adequate size, with cuff pressure measurement, if used, and to maintain the child's comfort to avoid tube movement and damage to the laryngeal and tracheal mucosa. The indication for tracheostomy in children is more related to the lack of perspective to resolve mechanical ventilation dependence.

In general, it is believed that there are no benefits of an early tracheostomy in patients with COVID-19. Since the mean time of mechanical ventilation in the patient with COVID-19 is approximately 21 days, many of these patients could be considered candidates for conversion to tracheostomy. On the other hand, the tracheostomy is a procedure considered an aerosol generator, representing an increased risk of transmission of SARS-CoV-2 to the surgical team and to the hospital environment through which the patient will move. Unlike droplets, which due to their weight and the effect of gravity have a limited transmission field, aerosols can remain in suspension for a long time and travel longer distances, with an increased risk of virus transmission. This occurs not only during the procedure, but also in the postoperative period, since the handling of a tracheostomy, with the need for frequent aspirations and the risk of decannulation with the need for repositioning, generates aerosols. Therefore, when considering carrying out the procedure, it is important to take into account patient severity, their prognosis and the risk of care team contamination, which are crucial for fighting the pandemic.

Therefore, we suggest avoiding the elective tracheostomy whenever possible in a patient with COVID-19. When tracheostomy is considered necessary, the following are recommended: avoid using an electric or ultrasonic scalpel as they may favor the formation of aerosols; do not use ventilation rooms with positive pressure, as they favor the dispersion of aerosols; whenever possible, use closed-circuit suction systems and antiviral filter and operating rooms with negative pressure. In their absence, use rooms with normal pressure and keep the doors closed; the surgical team must consist of the least possible number of professionals. In a patient with prolonged intubation, curarization is suggested, especially when removing the tube and placing the tracheostomy cannula, to minimize the risk of coughing, which promotes aerosolization. Another care procedure suggested by the Brazilian Society of Thoracic Surgery^18^ is the discontinuation of mechanical ventilation, deflating the tracheal tube cuff and its disconnection from the ventilation system BEFORE the tracheal incision. After insertion of the tracheostomy cannula and cuff insufflation, the mechanical ventilation system can be connected, and ventilation restarted.

The care and handling of the tracheostomy cannula, such as aspiration and strap changes, particularly in children to avoid obstructions, should be carried out with all the above mentioned PPE, while there is a risk of COVID-19 contamination. The agility in this information can facilitate discharge as early as possible during the pandemic period. It is suggested, during the pandemic period, reducing the frequency of changes to a minimum, and for that, it is necessary to provide guidance to caregivers about warning signs for changes and when to seek in-person care.

## About the use of telemedicine

The regulation of Telemedicine in Brazil during the COVID-19 pandemic has been influenced by Ordinance N. 188 of February 3, 2020 of the Ministry of Health, declaring a Public Health Emergency of National Importance (ESPIN) as a result of COVID-19, Legislative Decree N. 6 of March 20, 2020 of the National Congress recognizing the State of Public Calamity with effect until December 31, 2020, Letter N. 1756/2020 of March 19, 2020 of the Federal Council of Medicine recognizing the possibility and ethics of using Telemedicine as an exceptional situation and while the measures to fight COVID-19 last, and Ordinance N. 467 of March 20, 2020 of the Ministry of Health regulating Telemedicine actions as a means of fighting the ESPIN as a result of COVID-19.^20^ Similarly, considering that during this pandemic period, the high incidence and prevalence of otorhinolaryngological diseases remains, but there is a need to reduce physical contact between doctors and patients (without impairing the necessary care for adequate assistance) and the need to reduce the circulation of people.

Therefore, at the present time, there is the possibility of wide and comprehensive use of Telemedicine, including Teleguidance, Telemonitoring, Teleinterconsultation and Teleconsultation, aiming to a complete and humane care for isolated patients or those unable to have physical/face-to-face access to the doctor, with total professional autonomy and discretionary judgment regarding the form, method and content of care/treatment, aiming at broad health care and life protection, increased by the current state of necessity.

We recommend obtaining the adequate express authorization from the patient or legal advisors to use non-face-to-face assistance through Telemedicine, explaining the method limitations related to not performing the full physical examination. The express authorization can be obtained by recorded video, written message or signature of a specific consent form made available by ABORL-CCF. Special care should also be taken with the storage, transmission and use of patient data, respecting the ethical and legal responsibilities of confidentiality and professional secrecy, including the use of technological tools that guarantee this protection. There is the possibility of providing assistance and care by means of “online” (synchronous) or “offline” (asynchronous, in case of greater need) consultations, aiming at the most effective way of protecting the patient's health and life. It is essential to properly record medical appointments in the patient's medical record (electronic or physical), even if the appointments are recorded. It also emphasizes that there is no obligation to record consultations.

We advise that an alternative to face-to-face assessment be offered in a timely manner in cases where the limitation of an incomplete physical examination may increase the risk of an incorrect diagnosis. Moreover, logistic services can be used to send medical prescriptions and certificates or provide medical prescriptions and certificates in digital format with electronic signature through ICP-Brazil certificates. Charging for distance healthcare is allowed and remote care is also allowed for new patients without a diagnosis of COVID-19. We recommend not affiliating with intermediary companies (websites or applications) with a suspicious character, which may end up unscrupulously exploring and demeaning medical work. It is important to note that these determinations and authorizations are of an exceptional and transitory nature.

## Final considerations

The recommendations contained in this publication reflect the acquired knowledge and the scarce evidence about COVID-19 to date. If new evidence emerges that justifies a change in conduct, this publication may be updated.

## Conflicts of interest

The authors declare no conflicts of interest.
